# Risk factors for incomplete vaccination and missed opportunity for immunization in rural Mozambique

**DOI:** 10.1186/1471-2458-8-161

**Published:** 2008-05-16

**Authors:** Jagrati V Jani, Caroline De Schacht, Ilesh V Jani, Gunnar Bjune

**Affiliations:** 1 Department of Immunology, Instituto Nacional de Saúde, Maputo, Mozambique; 2 Department of General Practice and Community Medicine, University of Oslo, Norway

## Abstract

**Background:**

Inadequate levels of immunization against childhood diseases remain a significant public health problem in resource-poor areas of the globe. Nonetheless, the reasons for incomplete vaccination and non-uptake of immunization services are poorly understood. This study aimed at finding out the reasons for non-vaccination and the magnitude of missed opportunities for vaccination in children less than two years of age in a rural area in southern Mozambique.

**Methods:**

Mothers of children under two years of age (N = 668) were interviewed in a cross-sectional study. The Road-to-Health card was utilized to check for completeness and correctness of vaccination schedule as well as for identifying the appropriate use of all available opportunities for vaccination. The chi-square test and the logistic regression were used for statistical analysis.

**Results:**

We found that 28.2% of the children had not completed the vaccination program by two years of age, 25.7% had experienced a missed opportunity for vaccination and 14.9% were incorrectly vaccinated. Reasons for incomplete vaccination were associated with accessibility to the vaccination sites, no schooling of mothers and children born at home or outside Mozambique.

**Conclusion:**

Efforts to increase vaccination coverage should take into account factors that contribute to the incomplete vaccination status of children. Missed opportunities for vaccination and incorrect vaccination need to be avoided in order to increase the vaccine coverage for those clients that reach the health facility, specially in those countries where health services do not have 100% of coverage.

## Background

The prevention of child mortality through immunization is one of the most cost-effective public interventions in use in resource-poor settings. The Expanded Program on Immunization (EPI) aims at delivering the primary immunization series to at least 90% of infants [1]. However, inadequate levels of immunization against childhood diseases remain a significant public health problem in resource-poor areas of the globe [2]. Nonetheless, the reasons for incomplete vaccination and non-uptake of immunization services are poorly understood.

In Mozambique, the EPI targets seven diseases, namely tuberculosis, poliomyelitis, diphtheria, pertussis, tetanus, hepatitis B and measles. The reported coverage of the basic EPI vaccines in Mozambique is 80%–95%, but these figures include incomplete and incorrect vaccinations [3,4]. This may be one of the reasons why measles epidemics continue to occur in Mozambique despite of the reported 80% measles vaccination coverage [5]. Additionally, it has also been shown that immunization coverage in not uniform throughout the country, with rural areas presenting significantly lower coverage's [2] and, thus, contributing to the circulation of wild-type measles.

The effectiveness of immunization programs in resource-poor settings can be influenced by factors such as the coverage of the health network, the existence and quality of outreach services, the quality of the cold chain, the liaison of communities with health services, the existence of population movements, and several other factors that are related to the vaccines in use, to health services or to communities. The relative effect of each one of the above factors may significantly vary according to geographical areas [6]. In this context, the understanding of local hurdles for effective immunization programs is crucial to develop and implement appropriate solutions.

This study was carried out in a rural setting in southern Mozambique with the following aims: *first*, to determine the magnitude of incorrect vaccination figures reported amongst children less than two years old; *second*, to identify factors that adversely influenced vaccination completeness and contributed to missed opportunities for the immunization of children.

## Methods

### Study area

This study was carried out in the district of Magude, located in southern Mozambique. The district has a total area of 6,597 Km^2 ^and is divided into five administrative posts (Magude Village, Motaze, Mapulanguene, Panjane, and Mahele). The total population of the district is 42,788 inhabitants and its local economy is based on subsistence agriculture, cattle-grazing and hunting. Between 1976 and 1992, the civil war in Mozambique lead to the migration of a large proportion of the population to urban centres in Mozambique or to South Africa. Re-settlement of refugees in the district initiated in 1992, with 75% of all the returnees initially settling in the Magude Village [7]. The districts' monthly health reports indicate a coverage of 75% for all vaccines.

### Study design

A cross sectional survey was conducted in all five administrative posts of the district between the 1^st ^of July and 30^th ^of August 2001. The completeness and correctness of vaccination schedules were checked using standardised questionnaires. Factors leading to missed opportunities or incompleteness of vaccination were also searched. The child's vaccination dates, number of doses and dates of other visits to the health facility were extracted from the child's Road-to-Health Card (RHC). Information about migration history, mother's knowledge on immunization and the program, and accessibility to the nearest health facility was obtained through verbal information. The data collectors for the surveys were recruited among women in the Magude Village and were all able to speak and write Portuguese (national language) and Xichangana (local language).

### Study population and sampling

Mothers with children younger than twenty four months (as per the reported dates of birth), who lived in the area for at least nine months prior to the survey, and who possessed the child RHC were included in the study. The mobilisation of respondents for the survey was performed in a one week period before the inquiries. The traditional leaders (*régulos*) were instrumental in helping with the recruitment process. To ensure that most of families were at home during the time of the survey, prior information was given to the *régulos *about the dates of survey.

All clusters of populations (*quarteirão*) in the administrative post of Magude Village (26 clusters) were selected for the survey. From the other administrative posts only some clusters were randomly chosen because of time and resource constraints. In Magude Village, the sampling started from the geographic centre of the cluster. The direction of data collection was randomly chosen with the toss of a coin and the numbers of houses lying in that direction until the boundary of the cluster were counted. A number between one and *X *was randomly chosen, with the survey beginning at the corresponding house and including every second house thereafter. In the remaining administrative posts, which were more dispersed, the clusters covered a large area and a different procedure of selection was adopted. The chief of the administrative post informed the researcher of the numbers of clusters and households in that post and some of these clusters were chosen randomly. In the selected cluster, a direction was randomly chosen and every house in that direction was selected for the survey.

This study was approved by the Mozambican Bioethical Committee and the Regional Ethical committee in Western Norway. Informed consent was obtained from the mothers through their signature or finger prints after explaining the aims of the study. All included mothers were free to withdraw from the study according to their convenience at any time without any repercussions. There were no refusals to be enrolled in the study.

### Definitions and statistics

The questionnaires were coded, data was entered using the EPI INFO 6.0 programme and later converted to SPSS (version 16.0). The correct intervals for immunization were calculated comparing the dates of vaccination with the date of birth. The child was "correctly vaccinated" if it had a BCG scar and had received all the EPI vaccines within the minimum intervals of time as specified by national policy: DTP/OPV first dose not before six weeks of age with an interval of the least four weeks between doses and measles vaccine not before nine months of age. If a child came to a health facility or outreach site, and did not receive the vaccination for which he or she was eligible, this was considered to be a "missed opportunity" for vaccination. The accessibility to a health facility with immunization facilities was measured according to mothers' verbal information on impression of the distance, time spent to reach the nearest vaccination site and the money spent on transport. Migration history was based on verbal information of prior movement of mothers from one place of dwelling to another over the last two years. Religious believer was considered if the mother practiced any religion and the mother's schooling was considered independent of the number of years at school.

Proportions were used to describe the study population and to estimate vaccination status and missed opportunities for immunization. Differences in proportions were calculated using the chi-square test with at 5% of significance level. The ANOVA test was used to compare mean values among subclasses. Associations between factors and missed opportunities or incomplete vaccination status were tested first by the chi-square test. In order to investigate relative importance of the variables in relation to the dependent factors and any confounding between them, they were fitted together in a binary logistic regression model. The cluster sampling design was taken into account during the analysis (SPSS version 16.0).

## Results

Seven hundred houses with children younger than two-year old were visited. In total, 668 children fulfilled the inclusion criteria. The mean age of the children was 13 months (range 2–24) and 333 (49.8%) were of the female gender. Only 32/668 mothers (4.7%) could not produce a RHC or had been not living in the area for the last nine months and were later excluded from the sample.

The survey showed that 348 mothers (52.0%) lived far away from the health facility. The average walking time to the nearest health facility was one hour (range 3 minutes – 4 hours). Those who needed transport, spent in average 18.0 Meticais (range 1.0 – 140.0; 1 USD = 18.0 Meticais) for a one way trip to the health facility. The majority (n = 538, 80.5%) of the mothers were born in the Magude district and only 29 (4.3%) had a migration history in the past two years. Among them, 17 had migrated to an area within the district, 11 to places outside the district but within Mozambique, and just one to a country outside of Mozambique.

Only 142 (21.2%) mothers had heard about the EPI as a specific program. The major sources of information for this knowledge were the health facility (n = 67, 47.1%), the radio (n = 44, 30.9%), community workers (n = 25, 17.6%) and family and friends (n = 14, 9.8%). Vaccination was considered to be important by 642 mothers (96.1%) while 606 mothers (90.7%) did not know any contra-indication for immunization. However, only 423 (63.3%) knew that the vaccination program should be finished at the age of nine months with the measles vaccine.

The RHC showed that 507 (75.8%) children had been born in a health facility while nine (1.3%) children had no information registered regarding their place of birth. Among the 152 mothers that had a home delivery, 43 (28.2%) had delivered alone without any type of help, 102 (67.1%) had help from family or neighbours, and five (3.2%) were assisted by a traditional birth attendant.

### Assessment of immunization status of children and risk factors associated with incomplete vaccination

Almost three quarters of the children (n = 479, 71.7%) had a complete vaccination status. Specific reasons for not being able to vaccinate their child were identified by 185/189 (97.8%) mothers. These were: a) Reasons associated with health services delivery (n = 71, 38.3%), including long waiting time, no personnel at the health facility, no vaccines available on the day, no information about the day for vaccination and no vaccination given due to child sickness; b) Forgetting the day of immunization (n = 33, 17.8%); c) Difficulties in accessing the health facility (n = 29, 15.6%); d) Mother's sickness on the day of vaccination (n = 7, 3.7%); e) Migration (n = 4, 2.1%); f) Concomitant treatment by a traditional healer (n = 3, 1.6%); g) Other miscellaneous reasons (20.5%). Factors such as mothers' age, marital status, schooling level, migration history, gender of the child, understanding of the importance of vaccination, and migration history of the mother showed no significant differences with respect to children with complete and incomplete vaccination status.

Variables that showed significant differences between children with complete and incomplete vaccination status are depicted in Table 1. Variables that lost their statistical significance when included in the multi regression model were considered confounders for incomplete vaccination status of the children, and included: accessibility in terms of distance (P = 0.69) and need of transport (P = 0.11), mothers' knowledge about vaccination contra indication (P = 0.41) and religious beliefs (P = 0.36).

**Table 1:** Factors associated with incomplete vaccination status in children less than two years old, Magude District, 2000.

			**Bivariate analysis**		**Binary logistic regression analyses**	
			
**Variable**	**Completely vaccinated N = 479 (%)**	**Incompletely vaccinated N = 189 (%)**	**OR (95% CI)**	**P value**	OR* (95% CI)	**P value**
**Administrative posts**						
Magude village	392 (81.8)	104 (55.0)	1	<0.001	1	<0.001
Out of Magude village	85 (17.7)	84 (44.4)	3.72 (2.53–5.49)		2.89 (1.80–4.64)	
**Distance to the HF**						
Near	260 (54.2)	58 (30.6)	1	<0.001		
Far away	219 (45.7)	129 (68.2)	2.64 (1.82–3.84)		1	
**Time to arrive to HF **^&^	N = 281	N = 95				
	53.6 (SD 41)	84.64 (SD 55.6)		<0.001	3.64 (1.71–7.74)	0.001
**Need of transport to arrive to the HF**						
Yes	216 (45.0)	128 (67.7)	1	<0.001		
No	252 (52.6)	54 (28.5)	0.36 (0.25–0.53)			
**Mother's schooling**						
Yes	264 (55.1)	51 (26.9)	1	<0.001	1	0.001
No	207 (43.2)	135 (71.4)	3.38 (2.30–4.97)		2.24 (1.41–3.56)	
**Knowledge about vaccine contraindication**						
Yes	25 (5.2)	22 (11.6)	1	0.003		
No	442 (92.2)	164 (86.7)	0.42 (0.22–0.80)			
**EPI information**						
Yes	123 (25.6)	19 (10.0)	1	<0.001		
No	352 (73.4)	169 (89.4)	3.11 (1.81–5.40)		2.02 (1.19–3.42)	0.009
**Mothers pro vaccination**						
Yes	466 (97.2)	176 (93.1)	1	0.005	1	0.001
No	10 (2.0)	12 (6.3)	3.18 (1.26–8.09)		2.92 (1.51–5.62)	
**Religious beliefs**						
Yes	313 (65.3)	101 (53.4)	1	0.004		
No	162 (33.8)	86 (45.5)	1.65 (1.15–2.36)			
**Child born**						
Inside of Mozambique	465 (97.0)	172 (91.0)	1	<0.001	1	
outside of Mozambique	10 (2.0)	16 (8.4)	4.33 (1.81–10.47)		5.20 (3.35–11.51)	<0.001
**Place of delivery**						
HF delivery	384 (80.1)	123 (65.0)	1	<0.001	1	0.03
Home delivery	88 (18.3)	64 (33.8)	2.27 (1.52–3.38)		1.78 (1.28 – 3.36)	
**Child age^&^**						
	13 (SD 6)	11.24 (SD 6.4)		<0.001	1	0.01
					0.94 (0.90–0.99)	

The table presents estimated odds ratio (OR), with 95% confidence intervals (CI), for selected variables found to be associated with incomplete vaccination status (P < 0.05) in bivariate analyses and adjusted odds ratios using the regression model to identify the risk factors for incomplete vaccination status for children less than two years old.

OR – Odds ratio, OR* – Adjusted odds ratio, % – percentage, *1 = reference variable, HF – health facility, C.I. – coefficient interval, EPI – Expanded Program on Immunization.

& – Mean values compared using Anova test.

Only significantly associated variables (p < 0.05) are shown in the table.

### Assessment of vaccination schedule

Incorrect vaccination was identified in 100/668 (14.9%) children and included: absence of BCG scar in 60 children; receiving two doses of the DTP vaccine in the same day in 16 children; receiving the DTP vaccine with less than four weeks interval in four infants; receiving the measles vaccine before 9 months of age in 30 subjects. Eleven children had more than one mistake identified in their immunization schedule.

### Assessment of missed opportunities for vaccination and associated risk factors

Missed opportunities for vaccination were found in 172 (25.7%) children. The mean number of missed opportunities for vaccination per child was 1.73. Among the children with missed opportunities for vaccination, 26 (15.1%) had more than one missed opportunity for vaccination (range 1–15) while 36 (20.9%) of the children could have completed their vaccination program if they had not missed the opportunity for measles vaccination. Children with missed opportunities for vaccination were more likely to have an incomplete vaccination status than children without missed opportunities P < 0.001 (see Table 1).

Only 24 (13.9%) of the mothers could recall the reason for the missed opportunities: nine (37.5%) had a sick child, two (8.3%) were not aware of the need for immunization and 13 (54.1%) referred a lack of vaccines availability in the health facility. The variables contributing to a significant difference between children with and without missed opportunities for vaccination are shown in Table 2. Only one variable, EPI information (P = 0.09), lost its statistical significance when included in the multi regression model.

**Table 2:** Factors associated with missed opportunity for vaccination in children less than two years old, Magude District, 2000.

			**Bivariate analysis**		**Binary logistic regression analyses**	
			
**Variable**	**MO N = 172 (%)**	**No MO N = 473 (%)**	**OR (95% CI)**	**P value**	**OR (95% CI)**	**P value**
**Child born**						
Outside of Mozambique	14 (8.1)	12 (2.5)	3.47 (1.48–8.20)	0.001	0.26 (0.12–0.54)	<0.001
Inside of Mozambique	155 (90.1)	461 (97.4)	1		1	
**Place of delivery**						
HF	143 (83.1)	346 (73.1)	2.05 (1.25–3.38)	0.002	2.29 (1.37–3.83)	0.02
Home	25 (14.5)	124 (26.2)	1		1	
**EPI information**						
Yes	46 (26.7)	89 (18.8)	1.03 (1.03–2.43)	0.028		
No	125 (72.6)	382 (80.7)	1			
**Civil status of the mother**						
Single, divorced and widow	46 (26.7)	88(18.6)	1.60 (1.04–2.45)	0.024	1.68 (1.07–2.64)	0.02
Married and conjugal living	126(73.2)	385(81.3)	1			

The table presents estimated odds ratios (OR*), with 95% confidence intervals (CI), for selected variables found to be associated with missed opportunity for vaccination (P < 0.05) in bivariate analyses and adjusted odds ratios using the regression model to identify the risk factors for missed opportunity for vaccination in children under two years old.

MO – Missed opportunity for vaccination, OR – Odds ratio, OR* – Adjusted odds ratio, % – percentage,

*1 = reference variable, HF – health facility, C.I. – coefficient interval, EPI – Expanded Program on Immunization.

Only significantly associated variables (p < 0.05) are shown in the table.

## Discussion

This study was conducted in a homogeneous rural community in southern Mozambique. The majority of the interviewed mothers were from low or medium social economical status and all of them had a low or no educational level [8]. Only children with a RHC were included in our survey. As these comprised more than 95% of the interviewed population, it is unlikely that our results are biased by excluding children with no RHC.

Our results show that mothers are motivated, understand the benefits of immunization and are willing to walk long distances to benefit from health care. In Mozambique, the average monthly salary is 540 MT (USD 33). Many of the mothers in the Magude District spent an average of USD 2.0 per trip to the health facility. Therefore, just the direct travelling costs for obtaining all the EPI vaccines are approximately USD 10.0 per child, the equivalent to 2.5% of the average annual salary. As the majority of the mothers were peasant farmers with no formal income, the money for the travelling costs came from other people in the family or alternative sources[8]. It was observed that in the administrative posts other than Magude Village, the risk of incomplete vaccination status was high. The reasons for this are probably linked with difficult access to the health facility since the population settlements were more dispersed.

Accessibility as a function of distance and need for using transport were identified as confounder variables for immunization non-uptake. However, spending longer than 60 minutes to reach the nearest health facility was demonstrated to have a strong negative influence in immunization uptake. Accessibility was seen to have no association with missed opportunities for vaccination which appeared to be linked to the quality of health facility services [9]. A friendly organisation of the health facility and a good coordination between fixed and outreach activities, including a strong involvement of the local community, could help to decrease the mothers' expenses on transportation and the time spent for obtaining vaccination services.

In spite of the fact that Magude district was considered a high migration area [8], our results show that only 4% of our population had a history of migration. Only four mothers referred to migration as a cause of non completion of their children's vaccination schedule. In a previous study in Mozambique [10], migrants were found to be less well vaccinated compared to long term residents in the area. In Cameroon, children of mothers with a history of migration were seen to be less vaccinated [11]. Migration might also be associated with low vaccine uptake due to the weak social integration of migrating populations. Nevertheless, factors such as the vaccination coverage in the settlement area and the mother's awareness of the importance of immunization may also play an important role.

There was no evidence to support that child gender had any impact on vaccine uptake or in defining missed opportunities for vaccination in our study area. In some societies with cultural discrimination against female children, boys have a greater chance to be vaccinated [12]. Marital status and age of the mothers were not seen to be associated with the use of immunization services. In other settings, both younger [13] and older age of mothers [12] has been reported to be associated with incomplete vaccination.

The low educational level of mothers has been previously associated with low vaccine uptake [13,14]. In the present study, the no schooling status in mothers was strongly associated with low vaccine uptake. However, no association was identified between schooling of mothers and missed opportunities for vaccination. Mothers' educational levels had no influence on the child's vaccination status, probably because very few mothers had more than primary school education.

Health workers were seen to be a potential source for disseminating information relating to the immunization program in this community. This emphasised their position as role models in the rural community. The strengthening of communication, education and information skills of the health providers is an important step for improving health services in general. Regardless of the difficult accessibility to a health facility, the majority of the mothers had a delivery assisted by a health worker. Still, the high proportion of home deliveries (22.7%) represents a significant public health concern, specially considering the limited role of traditional birth attendants in this community. Moreover, home delivered children have a 2.27 times higher risk of not completing their vaccination program. Hence, the increase in the proportion of deliveries within health facilities will also lead to a better effective coverage of the EPI.

Previous studies in Mozambique [10,15], have identified missed opportunities for vaccination and inappropriate use of contra indications as important factors inhibiting better EPI coverage.

In Magude District, we found that 28% of the children under two years of age were still in need of completing their vaccination program either due to incorrect vaccination or to missed opportunities. The high percentage of children without the BCG scar and vaccinated before nine months of age against measles is disturbing. Birth place outside Mozambique, change in immunization providers and living outside the Magude village were the factors that showed a stronger association with lower vaccination uptake.

Ninety one percent of the mothers did not know any contra indications for immunization. However 12% of 185 children had not been immunised at the health facility due to child illness. Minor illnesses in the family (fever, headache) had also been related with the non completeness of immunization program [16]. In this study, the mother's knowledge about any vaccine contraindication played a confounder role.

It has been reported in a review of 79 missed opportunity studies [9] that the quality of health services were an important cause of missed opportunities for vaccination. The large number of missed opportunities for vaccination in this community proved that the use of health services was high. Thus, exploiting visits for curative care would have been a cost-effective way of fully immunizing a child and increasing the EPI coverage. The all opportunities strategy is difficult to follow when the same health staff is part of both the fixed vaccination post and the mobile team. However, midwives and curative staff should be more involved in checking the vaccination cards and sending people to the immunization sessions. With scarce human resources and overwork, optimal co-ordination of different activities at fixed health stations and in mobile teams may almost be impossible without the strong involvement of the community.

## Conclusion

The utilization of health services by the community in the Magude District is good. If the missed opportunities for vaccination and incorrect vaccinations were avoided, the vaccine coverage could easily reach national targets. Migration of the mother or their educational levels, did not influence in the vaccination uptake or contribute to missed opportunities for vaccination. The mothers were seen to be in favor of immunizing their children and were well informed about the immunization program, but they faced many barriers to complete their children's vaccination program. Special attention should be paid to mothers with home delivery as a BCG at birth is the gateway into the EPI.

## Competing interests

The authors declare that they have no competing interests.

## Authors' contributions

JVJ and GB conceptualized the study. JVJ was responsible for data acquisition. JVJ, CDS and IVJ were responsible for analysis and interpretation of data. IVJ provided technical input, review and project direction. JVJ, CDS, IVJ, and GB collaboratively wrote the manuscript. All authors read and approved the final manuscript.

## Pre-publication history

The pre-publication history for this paper can be accessed here:


